# Translating, contextually adapting, and pilot testing of psychosocial and mental health assessment instruments for Congolese refugees in Rwanda and Uganda

**DOI:** 10.1186/s13031-022-00447-z

**Published:** 2022-04-15

**Authors:** Rosco Kasujja, Paul Bangirana, Anna Chiumento, Tasdik Hasan, Stefan Jansen, Daniel M. Kagabo, Maria Popa, Peter Ventevogel, Ross G. White

**Affiliations:** 1 Department of Mental Health and Community Psychology, Makerere University, Kampala, Uganda; 2 Department of Psychiatry, Makerere University, College of Health Sciences, Kampala, Uganda; 3 Department of Primary Care and Mental Health, University of Liverpool, Liverpool, UK; 4 Center for Mental Health, College of Medicine and Health Sciences, University of Rwanda, Kigali, Rwanda; 5 Mental Health & Community Psychology and Behaviour Research Group, College of Medicine and Health Sciences, University of Rwanda, Kigali, Rwanda; 6 Division of Resilience and Solutions, Public Health Section, United Nations High Commissioner for Refugees, Geneva, Switzerland; 7 School of Psychology, Queen’s University Belfast, David Keir Building, 8-30 Malone Road, Belfast, BT9 5BN UK

**Keywords:** Mental health and psychosocial support, Refugees, Humanitarian settings, Post-conflict, Assessment, Depression, Adaptation

## Abstract

**Background:**

Forcibly displaced people are at elevated risk of experiencing circumstances that can adversely impact on mental health. Culturally and contextually relevant tools to assess their mental health and psychosocial needs are essential to inform the development of appropriate interventions and investigate the effectiveness of such interventions.

**Methods:**

We conducted two related studies: (1) to translate and contextually adapt the Patient Health Questionnaire (PHQ-9), a measure of depressive symptomatology, along with assessment instruments measuring levels of daily stress (Checklist for Daily & Environmental Stressors; CDES), social capital (Shortened and Adapted Social Capital Assessment Tool; SASCAT) and perceived social support (Multidimensional Scale of Perceived Social Support; MSPSS) for use with Congolese refugees; (2) to conduct pilot testing of the assessment instruments (including cognitive interviewing about participants’ views of completing them) and a validation of the adapted PHQ-9 using a ‘known group’ approach by recruiting Congolese refugees from refugee settings in Rwanda (n = 100) and Uganda (n = 100).

**Results:**

Study 1 resulted in the translation and adaptation of the assessment instruments. No substantive adaptations were made to the SASCAT or MSPSS, while notable linguistic and contextual adaptations were made in both sites to the CDES and the PHQ-9. The cognitive interviewing conducted in Study 2 indicated that the adapted assessment instruments were generally well received by members of the refugee communities. Participants recruited on the basis that local informants adjudged them to have high levels of depressive symptoms had significantly higher PHQ-9 scores (M = 11.02; SD = 5.84) compared to those in the group adjudged to have low levels of depressive symptoms (M = 5.66; SD = 5.04). In both sites, the adapted versions of the PHQ-9 demonstrated concurrent validity via significant positive correlations with levels of daily stressors. Each of the four adapted assessment instruments demonstrated at least adequate levels of internal consistency in both sites.

**Conclusions:**

The adapted versions of the PHQ-9, CDES, SASCAT and MSPSS are appropriate for use amongst Congolese refugees in Rwanda and Uganda. We recommend further application of the approaches used in the current studies for contextually adapting other assessment instruments in humanitarian settings.

## Background

Eastern Democratic Republic of Congo (DRC) has experienced multiple interrelated conflicts for more than a quarter of a century [1, 2]. The ongoing violence and instability has led to an exodus of almost a million refugees and asylum seekers from the DRC to neighbouring countries [3]. In early 2020, Uganda and Rwanda were the first and fourth largest recipients of refugees from the DRC—hosting 422,989 and 76,266 refugees respectively [3]. Congolese refugees living in these neighbouring countries experience elevated levels of mental health difficulties [4, 5] associated with social circumstances in their country of exile and past experiences of violence and adversity [6, 7]. A key strategy for supporting the mental health and wellbeing of conflict-affected Congolese populations is to provide services within the community, and to strengthen existing social support systems that may have been weakened due to conflict and/or displacement [8–11].

High levels of mental health difficulties have consistently been recorded in areas affected by conflict. In a recent meta-analysis of studies among adults living in areas that had experienced conflict less than 10 years previously [12] estimated a point prevalence of mental disorders of 22.1%. Experiences of adversity can occur throughout the refugee cycle: in the country of origin, (e.g. exposure to violence, torture and persecution), during flight (including exploitation, impoverishment and lack of resources), as well as in displacement settings (e.g. stigma, discrimination, stress associated with asylum processes and lack of hope for the future) [13–15] Social adversity in the form of ‘daily stressors’ such as lack of access to basic resources, isolation, lack of safety and security, family violence, and communal tensions, can be an important determinant of common mental health issues [16]. Conflict-related traumatic events and displacement-related stressors may share a common putative causal mechanism in impacting the mental health and wellbeing of forcibly displaced communities by limiting a person’s freedom to engage in forms of behaviours which they value [17].

Having tools available to carefully assess important dimensions of the mental health and wellbeing of forcibly displaced people is important for the provision of humanitarian support. Standardised assessment instruments need to consider local cultural and linguistic contexts in order to be reliable and valid [18–20]. Assessment instruments that have been adapted for the local setting are more likely to assess the specific construct of interest appropriately [21].

The current paper reports on two related studies that form part of the same iterative process aimed at adapting, pilot testing and (in the case of the PHQ-9) rapidly validating assessment instruments. Study 1 reports on a procedure to translate and contextually adapt four assessment instruments for use with Congolese refugees in Rwanda and Uganda. These assessment instruments are being used in the *Community-based Sociotherapy Adapted for Refugees* (CoSTAR; http://www.liverpool.ac.uk/costar) project that aims to evaluate community-based sociotherapy as an intervention for reducing depressive symptomatology among Congolese refugees in Rwanda and Uganda. Study 2 reports on pilot testing of the adapted assessment instruments and a rapid validation of the Patient Health Questionnaire (PHQ-9) [22] which is the primary outcome measure in the CoSTAR project, and describes the outcome of cognitive interviews aimed at assessing the comprehensibility and the acceptability of all four translated assessment instruments in both sites, as well as an indication of the internal consistency of all four assessment instruments. We shall separately describe the methods and results of Study 1 and then the methods and results of Study 2, before jointly discussing the implications of the two studies.

## Study 1 (Translation and contextual adaptations of assessment instruments) Methods

### Settings

*Location 1*: Gihembe Refugee Camp in the Northern province of Rwanda is one of the oldest refugee camps in Rwanda. It currently hosts 12,206 refugees from DRC [23], almost all of whom (99%) are Kinyarwanda speaking Congolese survivors (and their offspring) of the 1997 massacre in the Mudende Refugee Camp in Western Rwanda [24]. The camp has approximately equal numbers of male and female refugees and an average family size of 4.5 people [25]. The majority of Gihembe refugees are Christian, with a minority Muslim population. The ‘Imiryango’ (clans) constitute a vital element in the camp’s socio-cultural ecosystem, contributing strongly to individual and family/social identities. Administratively, the camp is divided into 44 ‘villages’ clustered into 12 ‘quartiers’. Everyday life in the camp is overseen by an Executive Committee composed of eight members of the refugee community, facilitated by the Ministry of Emergency Management, and various humanitarian organizations provide support across all sectors including mental health and psychosocial support. Refugees in Rwanda are granted the right to work, start businesses, continue education, and are not subject to an encampment policy [26].

*Location 2*: Kyangwali is a refugee settlement in the Kikuube district of Western Uganda. It was established in the 1960s to accommodate refugees from Rwanda. Following the voluntary repatriation of most Rwandans in 1994, the settlement has predominantly hosted refugees from the DRC. As of January 2021, a total of 125,039 persons—53% female—lived in the 42,248 households of Kyangwali [27] spread across 28 different villages. Most refugees are from the DRC (120,328) with smaller populations from South Sudan (3439), Rwanda (1147) and other countries (125) [27]. With Uganda granting refugees the right to work, approximately half of the population in Kyangwali have an occupation, mostly in agriculture [27].

### Assessment instruments

*The Patient Health Questionnaire* (PHQ) [22] is a self-administered version of the Primary Care Evaluation of Mental Disorders diagnostic instrument for common mental disorders. The PHQ-9 [22] is the depression module, which scores each of the nine DSM-IV criteria on a scale of "0" (not at all) to "3" (nearly every day). The total score is calculated by summing the scores for each item (range: 0 to 27). The PHQ-9 also has a supplementary item that asks participants to reflect on ‘how difficult have these problems made it for you to do your work, take care of things at home, or get along with other people?’ (assessed on a four-point Likert scale ranging from ‘Not at all difficult’ to ‘Extremely difficult’). This item is not converted into a numerical score and hence is not included in the PHQ-9 total score. PHQ-9 scores ≥ 5, 10, 15, and 20 represent mild, moderate, moderately severe, and severe depression, respectively [28]. The initial validation of the diagnostic properties of the PHQ-9 was based on data from two studies involving 3,000 patients in seven obstetrics-gynaecology clinics and 3,000 patients in eight primary care clinics in the United States of America [29, 30]. The scale has been widely used as an outcome measurement for depression research and has been validated in various settings in Sub-Saharan Africa [31] including in Uganda and Rwanda [32, 33]. A recent study [34] conducted with South Sudanese refugees in Uganda found adequate internal consistency. A cut-off score of ≥ 10 has been proposed to be appropriate for screening for possible depression in East Africa [35, 36].

*Checklist for Daily & Environmental Stressors* (CDES) [37] is a 14-item checklist that assesses problems with attaining food, water, shelter, fair access to services, safety and protection, legal services, harassment by police, harassment by the local population, healthcare services, sanitation facilities, employment, education, freedom of movement, and feeling humiliated or disrespected. A total daily environmental stressors score can be calculated by the total sum of the number of stressors [37].

*The Shortened and Adapted Social Capital Assessment Tool* (SASCAT) [38] is a multifaceted instrument designed to collect social capital data at the household, community and organizational (e.g. non-governmental organizations) levels. The items focus on the opportunities and constraints that individuals and groups face by assessing the social assets and networks that determine their access to resources. The SASCAT and other social capital scales have been widely used, including in Africa, but are rarely adapted for the local context and validated [39]. A Kinyarwanda version of the scale, with some linguistic and contextual adaptations has been developed [40], and was subsequently used in community-based sociotherapy research in Rwanda [40, 41].

*The Multidimensional Scale of Perceived Social Support* (MSPSS) [42] is a brief measure of the perception of the support that a respondent receives from three sources: a significant other, family, and friends. The MSPSS was originally validated in western populations but has since been validated in South Asian populations [43] and in Uganda [44]. The internal consistency of the MSPSS has been shown to be adequate [45].

### Procedure

Table 1 describes the three-step process that was used to translate and contextually adapt the assessment instruments. This process was an adapted version of an approach originally developed by van Ommeren and colleagues [46].

**Table 1:** Procedure for linguistically translating and culturally adapting assessment instruments in the sites in Uganda and Rwanda

Step	Kyangwali, Uganda	Gihembe, Rwanda
A—Initial expert translation	Each of the instruments were translated from English to standard Swahili by a staff member at the Department of Languages at Makerere University, Kampala, Uganda.	Group translation from English by a team of six bilingual (Kinyarwanda-English) members of the CoSTAR research team. Team members noted items or concepts considered difficult to either understand and/or translate (e.g. terms and phrases that lacked obvious equivalents in standard Kinyarwanda). Proof-read by 4 external members fluent in Kinywarwanda.
B—Review by local mental health experts and practitioners inside and outside the refugee settings, and cultural-competent members of the CoSTAR Refugee Community Advisory Groups (RCAG) to assess comprehensibility, acceptability and relevance	The translated instrument produced in ‘Step A’ were reviewed and the standard Swahili translation of the instruments were appropriately amended to Congolese Swahili.	The translated instruments produced in ‘Step A’ (along with the associated notes) were reviewed and appropriate Kinyarwanda translations for phrasing and concepts that had been noted as challenging were proposed.
C—Instrument adaptation workshop (attendees included RCAG members, representatives of the MHPSS implementing non-governmental organization, Community Based Sociotherapy Rwanda staff, and members of the local CoSTAR research teams)	Data collected as part of a rapid qualitative study exploring the problems experienced by the communities and the tasks they undertake as part of their routine functioning was presented in Kinyarwanda (Rwanda) or Kiswahili (Uganda) to the workshop attendees (see Chiumento and colleagues (10) for details of this study). Two groups (both featuring bilingual speakers of Kinyarwanda/Congolese Kinyarwanda-English in Rwanda, and Congolese Swahili-English in Uganda) were formed from the workshop attendees. Each group focused on two different assessment instruments (Group 1: PHQ-9 & SASCAT; Group 2: MSPSS & CDES). The groups were provided with the local language versions of the assessment instruments produced in ‘Step B’ alongside the original English language versions. The groups reviewed and compared each of the items in the assessment instruments in the two language versions. Issues with the translation were recorded by a designated ‘scribe’ on a *Translation Monitoring Form* (46) which drew specific attention to: *Comprehensibility* (i.e. is the translation understandable in the local language?) *Acceptability* (i.e. would the respondent be uncomfortable honestly answering the question posed?) *Relevance* (i.e. is this question relevant in the local culture?), and *Completeness* (i.e. would translating the items back into English convey the same concepts and ideas as the original?) There was also space on the forms for the groups to record further comments if required. In addition, the group working on the CDES was asked if any items should be omitted, or additional items added considering the findings from our rapid qualitative study (10). Based on group consensus through the *Translation Monitoring Form* prompts, each group produced agreed translated versions of each assessment instrument. The English language versions of the assessment instruments were removed. Two members (including the scribe) from Group 1 presented the versions of the PHQ-9 and SASCAT to Group 2 by reading the items aloud and making the written copy available to view. This served as a cross-check for any issues relating to comprehensibility, acceptability, relevance and completeness. Notes about any additional changes that were agreed by consensus were made. Similarly, two members of Group 2 (including the scribe) presented the versions of the MSPSS and CDES to Group 1 by reading the items aloud and making the written copy available to view. Any additional amendments agreed by consensus were noted.	

### Ethics

Ethical approval for the collection of data in both Study 1 and Study 2 were provided by University of Rwanda Institutional Review Board, Makerere University School of Social Sciences Research Ethics Committee, and University of Liverpool Central Research Ethics Committee. Approvals were also provided by the Office of the Prime Minister (Uganda) to undertake the work in Kyangwali, and MINEMA (Rwanda) to conduct the work in Gihembe. All workshop participants were provided with Participant Information Forms and provided written informed consent prior to participating in research activities.

## Study 1 Results

No substantive adaptations were made in either site to the SASCAT or MSPSS, and there were no challenges of note with regard to the linguistic translation of the items of these assessment instruments. However, there were notable adaptations made in both sites on the CDES and the PHQ-9 to reflect the local refugee setting and linguistic nuances. Table 2 provides further details about these amendments.

**Table 2:** Adaptations to the CDES and the PHQ-9 in both sites

Assessment	Kyangwali, Uganda	Gihembe, Rwanda
CDES	Two new items were added to the CDES: ‘*Have a problem with income that often causes stress?’ (Una shida na mapato inakuleteya mafikiri?*) and *‘Have a problem with people not taking care of you?’ (Una shida nawatu wasiokujalia*?)	One new item was added to list of stressors assessed: ‘*Have a problem with firewood/fuel briquettes that often causes you stress?*’ (*Ese ufite ikibazo cy’ibicanwa kigutera kenshi guhangayika?*)
	Item 8, the phrasing of the CDES was changed from *'local population'* to *'people where you live'*	Item 8, the phrasing of the CDES was changed from *'local population'* to *'people where you live'*
PHQ-9	Item 2, the Congolese Swahili phrasing ‘*Feeling very bad about yourself or hopeless’* (*Uliwayi kujisikiya mubaya sana au kukata tama*) was used for ‘depressed’	Item 2, the Kinyarwanda phrasing ‘*disorder/illness of severe and persistent sadness*’ (*indwara y'agahinda gakabije*) was chosen to capture ‘feeling down/depressed’
		Item 7, ‘*Trouble concentrating on things such as reading newspaper and watching television*’ was amended to incorporate more relevant examples—specifically *‘listening to radio or caring for family’*

## Study 2 (Pilot testing of assessment instruments) Methods

In both Kyangwali and Gihembe, a cross-sectional survey of 100 adults (aged 18 years and above) was conducted to: (1) Facilitate cognitive interviews about the participants’ experience of completing the adapted versions of the four assessment instruments [46]. Participants completed all four assessment instruments, providing cognitive interview responses to one of these each—i.e. 25 participants providing feedback on each assessment instrument; (2) Examine the internal consistency of the four adapted versions of the assessment instruments in both sites; (3) Conduct a rapid validation of the adapted versions of the PHQ-9. To assess the construct validity of the PHQ-9, the ‘known groups’ approach was used (see: [47]). This is where local informants are invited to identify people on the basis of whether they were, or were not, presenting with difficulties that overlap with the particular signs and symptoms (in this case depressive symptomatology) assessed by the assessment instrument. Investigating whether there are differences between the groups can provide evidence of the construct validity of the adapted assessment instrument [47].

### Recruitment

Three *quartiers* in the Gihembe camp (out of 12) were selected as recruitment sites, and two villages (out of 28) in Kyangwali (Kagoma and Kasonga). To assist with the rapid validation of the PHQ-9, participants were purposively selected in both sites to include 50 individuals suspected of presenting with depressive symptomatology and 50 who were not. To identify and reach target groups, we consulted and engaged with local informants; mainly community leaders and community-health mobilizers in Gihembe, and a psychosocial officer working with *Humanitarian Initiative Just Relief Aid*, the organization that assisted with conducting the research activity in Kyangwali. Participants were purposefully recruited on the basis that the local informant was of the view that the person fell into one of two categories: (1) presenting with high levels of depressive symptomatology, or (2) presenting with low levels of depressive symptomatology. To ensure that depressive symptoms was consistently described, four items of the adapted versions of the PHQ-9 were used to guide the local informants’ assessment of potential participants. These included the two items that make up the PHQ-2 [48] (the first two items below) and an additional two items that assessed difficulties with sleep and reduced energy levels respectively. These additional two items were selected on the basis that they captured observable changes in the person’s ability to maintain established routines that can be characteristic of depressive symptomatology:

Item 1 of the PHQ-9: Little interest or pleasure in doing things (Congolese Kinyarwanda/Congolese Swahili; *Kudashishikarira ibyo ukora cyangwa ntushimishwe nabyo/Upungufu wa hamu au raha ya kufanya vitu).*

Item 2 of the PHQ-9: Feeling down, depressed, or hopeless (Congolese Kinyarwanda/Congolese Swahili; *Kumva wacitse intege, ufite agahinda,cyangwa nta cyizere/Uliwayi kujisikiya mubaya sana au kukata tamaa).*

Item 3 of the PHQ-9: Trouble falling or staying asleep, or sleeping too much (Congolese Kinyarwanda/Congolese Swahili; *Kubura ibitotsi, kubicikiriza hagati bikakugora kongera gusinzira, cyangwa gusinzira bikabije/Uliwayi kupata shida usingizi au kuweza kulala au kulala sana).*

Item 4 of the PHQ-9: Feeling tired or having little energy (Congolese Kinyarwanda/Congolese Swahili; *Kumva unaniwe cyangwa ufite intege nke/Uliwayi kujisikiya kuchoka au kutokuwa na nguvu).*

Care was taken to ensure that all those who were identified by informants as having high levels of depressive symptomatology were known to organisations providing Mental health and Psychosocial Support (MHPSS) in the two sites. Similarly, if members of the research team became aware that participants in either site required support from organisations acting as implementing partners for physical health and/or community protection these participants were appropriately supported to make contact with those organisations.

### Process for obtaining informed consent

Prior to consenting, an observational assessment of the potential participants’ mental capacity to consent was conducted by the research assistant. Written informed consent was obtained from all participants before participating in Study 2, following the reading aloud of the participant information sheet and answering of questions by a research assistant. For illiterate participants a witnessed line mark or mark (in lieu of a signature) was provided. In Gihembe, six potential participants identified by informants were excluded (two had complex mental health difficulties, three declined consent, and one was unavailable), all remaining participants approached provided consent. In Uganda, all the participants who were approached provided consent.

### Procedure

After participants provided informed consent, Research Assistants administered each of the assessment instruments to participants. The order of the administration of the assessment instruments was varied and balanced across locations and high/low depressive symptom groups. Each participant completed a cognitive interview in relation to the first assessment instrument they completed. The questions for the cognitive interviewing follow those used in a previous study [40]: (1) “Were there any questions that you found difficult to understand?”, “Which ones were they?”, “What was it that was difficult to understand?” (2) “Overall, how did you feel about completing this instrument? Please explain”. The research assistant posed the questions verbally in the local languages and the verbatim responses were recorded in writing in the local language. The responses were then translated by a bilingual member of the research team and independently checked for accuracy by another member of the research team.

### Analyses

The responses regarding the comprehensibility and acceptability of each of the measures were analyzed separately. Common themes emerging in the responses were noted and a frequency count of responses relating to each theme was made. For the rapid validation, descriptive statistics (e.g. means and standard deviations) were calculated. To assess criterion validity, between group comparisons (independent t-tests) were conducted to identify significant differences in the PHQ-9 scores between those whom informants adjudged to have *High Depressive Symptoms* vs. *Low Depressive Symptoms*. Building on previous research findings [37] that higher levels of daily stress (as assessed by the CDES) predicted higher levels of depressive symptomatology, bivariate correlations were conducted between the PHQ-9 and CDES to assess the convergent validity of these assessment instruments. Furthermore, bivariate correlations were conducted between the SASCAT and the MSPSS to assess the convergent validity of these measures. The criteria described used by Ursachi and colleagues [49] were used to ascertain the internal consistency of the adapted versions of each of the assessment instruments in both sites: Cronbach α of 0.6–0.7 indicates an acceptable level of reliability, between 0.7 and 0.8 a good level, and greater than 0.8 a very good level.

## Study 2 Results of cognitive interviewing

For each of the assessment instruments across the two sites, the vast majority of participants indicated that the adapted assessment instruments were clear and easy to understand. Most participants reported positive views about being asked about the issues that the instruments explore. A comparatively small proportion of participants reported that some questions upset them. A minority of participants also reported that they hoped the responses to such questions would lead to support being provided. The following section will highlight specific issues per site that were reported by two or more participants who completed the cognitive interviewing about a specific instrument. In the interest of brevity, we will summarise only feedback that highlighted potential problems in relation to particular items of the assessment instruments.

In Kyangwali, two participants highlighted that the CDES items about being harassed (i.e. by the police (item 7) and the local population (item 8)) were difficult to understand. With regard to the MSPSS, three participants in Kyangwali found item 10 of the MSPSS (i.e. There is a special person in my life who cares) difficult to understand. Two participants in Gihembe raised issues about the way in which the SASCAT made specific mention of ‘political groups’ (i.e. items 1 and 2) and ‘politicians’ (item 3) as response options (e.g. “*Yes, asking me about politics, I cannot discern what it is for, it is hard to understand it*.” Female, 39 years). These points seemed to be related to the fact that ‘political groups’ and ‘politicians’ were largely absent from the refugee setting, and some participants being wary about mentioning political groups and politicians given their experiences of forced displacement. Two participants in Gihembe also expressed confusion as to why items 1 and 2 of the SASCAT were interested to ask about their membership of groups more broadly (e.g. “*What is difficult to understand for me is the reason you want to know more about groups*” Female, 57 years). To address this, the following statement, which will be read by the assessor immediately before this question, has been added to the adapted versions of the SASCAT that will be used in the CoSTAR trial in both sites:

“*These are questions that try to capture where people may get support from and are asked all over the world. They include lots of different categories of responses that may be more or less relevant to your life in Gihembe/Kyangwali (delete as appropriate). The information we gather is useful to help us understand what sort of groups and associations are valuable in this community, and none of it will be shared outside of the research team. If you find a question sensitive, you are free to decline to answer and we will move onto the next question*”.

## Study 2 Results of rapid validation of the PHQ-9

Table 3 summarises the demographic characteristics of the samples. Of the 100 participants recruited in Gihembe 72 were females, and the mean age was 42 years (SD = 17.10). A total of 26 clans were represented, most commonly Abanyiginya (n = 12), Abasinga (n = 12), Abaha (n = 11), Abega (n = 10), and Abacyaba (n = 9). Of the 100 participants recruited in Kyangwali, 65 were male, with a mean age of 37 yrs (SD = 11.54). A total of 17 ethnic groups were represented in the Kyangwali sample—most commonly Nyabwisha (n = 58), Tutsi (n = 7), Gegere (n = 6), Hutu (n = 5), and Hima (n = 3). In both sites, the most commonly reported responses for marital status, education and employment status were ‘married’, ‘no schooling’, and ‘unemployed’ respectively.

**Table 3:** Demographic characteristics (%) of the sample for rapid validation

	Gihembe Rwanda			Kyangwali Uganda		
	Full Sample (n = 100)	Adjudged low levels of depressive symptoms (n = 50)	Adjudged high levels of depressive symptoms (n = 50)	Full Sample (n = 100)	Adjudged low levels of depressive symptoms (n = 51)	Adjudged high levels of depressive symptoms (n = 49)
Age	42 (17.11)	44 (18.91)	40 (15.04)	37 (11.37)	34 (11.54)	39 (10.65)
Years in camp	22 (2.49)	22 (3.24)	22 (1.49)	8 (6.79)	8 (6.52)	8 (7.11)
Sex						
Female	72	28	44	65	34	31
Male	28	22	6	35	17	18
Marital Status						
Cohabiting	1	1	0	1	1	0
Currently Married	41	28	13	53	28	25
Divorced	1	0	1	1	0	1
Never married	27	15	12	12	6	6
Separated	16	0	16	11	4	7
Widowed	14	6	8	22	12	10
Highest level of Education						
No Schooling	40	20	20	37	19	18
Primary school	25	8	17	13	15	21
Junior High School	16	5	11	36	8	5
Senior High School	17	15	2	5	4	1
College/Diploma	0	0	0	6	2	4
University	2	2	0	3	3	0
Occupation						
Evangelist/Pastor	2	2	0	1	0	1
Farming	1	1	0	20	6	14
Keeping house/homemaker	35	18	17	10	7	3
Paid work	2	2	0	3	2	1
Part-time jobs	4	3	1	0	0	0
Self-employed	15	8	7	8	3	5
Student	6	2	4	1	1	0
Unemployed	35	14	21	57	32	25

Results presented in Table 4 show that individuals who were recruited because they were thought to have high levels of depressive symptoms had significantly higher PHQ-9 scores (Gihembe: M = 11.02; SD = 5.84; Kyangwali: M = 12.26; SD = 7.04) compared to those in the group adjudged to have low levels of depressive symptoms (Gihembe: M = 5.66; SD = 5.04; Kyangwali: M = 8.90; SD = 5.41). The mean PHQ-9 score for the full sample in Gihembe was 8.34 (SD = 6.06), and in Kyangwali it was 10.55 (SD = 6.46).

**Table 4:** Descriptive statistics for the PHQ-9 responses in the two groups recruited on the basis of being adjudged to have high vs. low depressive symptoms in Gihembe and Kyangwali

	Gihembe, Rwanda					Kyangwali, Uganda				
	PHQ-9 Mean (SD)	PHQ-9: How difficult the problems have been for the participant (n)				PHQ-9 Mean (SD)	PHQ-9: How difficult the problems have been for the participant (n)			
		Not difficult at all	Somewhat difficult	Very difficult	Extremely difficult		Not difficult at all	Somewhat difficult	Very difficult	Extremely difficult
Full Sample (n = 100)	8.34 (6.06)	32	26	33	9	10.55 (6.46)	18	31	26	25
Low depressive symptoms	5.66 (5.04) N = 50	50	24	20	6	8.90 (5.41) N = 51	11	12	11	12
High depressive symptoms	11.02 (5.84) N = 50	14	28	46	12	12.26 (7.04) N = 51	15	13	15	13
t-value (df)	− 4.91 (98)					− 2.68 (98)				
p	*p* < 0.001***					*p* < 0.01**				

In both sites, the adapted versions of the PHQ-9 had significant positive correlations with the level of daily stressors as assessed by the adapted versions of the CDES (r = 0.36, *p* < 0.001 in Gihembe; r = 0.26, *p* < 0.05 in Kyangwali). Table 5 provides details of the correlations between the SASCAT total score and the MSPSS scores. In both sites there were significant positive correlations between all of these variables.

**Table 5:** Bivariate correlations and internal consistency scores for the SASCAT and MSPSS

	SASCAT Total score	
	Gihembe	Kyangwali
MSPSSTotal Mean	0.43***	0.29**
MSPSS Sign. Other Mean	0.34**	0.27**
MSPSS Family Mean	0.40***	0.22*
MSPSS Friends Mean	0.33***	0.30**

*p < .05; **p < .01; ***p < .001

Table 6 provides details of the internal consistency scores for the assessment instruments in both study sites. The PHQ-9 and MSPSS demonstrated very good levels of internal consistency in both sites, the SACAT good levels in both sites, and the CDES a good level in Kyangwali and an acceptable level in Gihembe.

**Table 6:** Internal consistency (Cronbach’s α) of the adapted versions of the measure

	Site	
	Gihembe	Kyangwali
PHQ-9	0.83	0.88
CDES	0.60	0.70
SASCAT Total score	0.78	0.70
MSPSS Total Mean	0.90	0.91
MSPSS Significant Other Mean	0.83	0.80
MSPSS Family Mean	0.87	0.83
MSPSS Friends Mean	0.85	0.78

## Discussion

Elevated levels of depressive symptomology have been reported amongst refugees from East African countries residing in settlements in the region [50–52]. The current studies sought to contextually adapt and validate the PHQ-9 measure of depressive symptomatology for use with Congolese refugees in Rwanda and Uganda. Study 1 sought to translate and contextually adapt four assessment instruments (PHQ-9; CDES, SASCAT and MSPSS) measuring depressive symptomology, daily stressors, social capital and perceived social support respectively. Study 2 sought to conduct pilot testing of the assessment instruments that included cognitive interviews with members of the refugee communities in both Gihembe and Kyangwali about their experience of being administered the assessment instruments, ascertain the internal consistency of the assessment instruments, and conduct a rapid validation of the PHQ-9.

The approaches used to translate and adapt the assessment instruments in the current study share similarities, but also some important distinctions with approaches used in other studies, which vary in the steps they take and the extent to which they include findings from formative research, local community involvement, piloting and validation, and forward and backward translation [40, 53]. As with the current study, Tol and colleagues’ [53] approach was based on the process developed by van Ommeren and colleagues [46]. The current study did not use formal back-translation, opting instead for a more streamlined pragmatic approach. In recent years, alternative approaches have been proposed based on *skopostheorie* [54] (skopos = purpose) which is a functional approach to translation defined as; ‘an area of translation theory that asserts that translational activity should be ultimately grounded in the purpose of the translation rather than the objective equivalency of the source and target texts’ [55]. Evidence suggests that *skopostheorie*-based approaches can yield higher quality translations in terms of both coherence, fidelity and usability in the specific setting [55]. Brislin [56] proposed back-translation as a key step for checking the fidelity of the translation. However, critics have highlighted that in back-translation the ‘equivalence’ of concepts cannot be guaranteed because mental health related terms are very difficult to translate directly [57, 58]. Furthermore, it has been highlighted that back-translation serves to prioritise the legitimacy of the source text and denigrates the worldview and context of the target population [55]. Jabir [59] proposed that the approach taken to translation should be guided by the purpose that the text was intended serves, rather than either the syntactic structure or the communicative effect of what is being translated. The process of back-translation has important time, human resource (including the availability of proficient translators), and cost implications—all of which may be in short-supply in humanitarian contexts. In the current study we invited members of the local Refugee Community Advisory Group to play an active role in the translation process. The involvement of ‘experts by experience’ in this way, provides an important opportunity to ensure that important issues are addressed during the translation process and the resulting assessment instrument resonates with the lived experience of those living in the research setting. In addition, this form of participant involvement is consistent with efforts to empower local community members to be more active in the research process. We propose that the approach used in the current study is a viable alternative that should be considered in such circumstances.

Results from the cognitive interviewing indicated high levels of acceptability and understandability across both sites. It was striking how frequently participants either (a) recognised the value of simply being asked about how they are doing; and/or (b) expressed hope for subsequent action and advocacy from the researchers to improve their situation. This has important implications for similar research being conducted in humanitarian settings. It is clear that asking questions exploring issues covered by the assessment instruments has the potential to raise hope in some, albeit not all, participants. Equally, though, there is an ethical quandary in relation to the risk that participants’ *hopes* for change become *expectations* for change, and what effect a failure to deliver on these expectations might have for both a given study and subsequent research conducted in the same population. It is therefore incumbent upon researchers to clearly communicate the limits of their involvement with participants and articulate what the research can hope to reasonable achieve, and what it cannot affect, as was done in this study.

Whilst there were a small number of items across the four measures that provoked negative reactions from participants in both sites, these tended to be isolated reactions. Most often these related to items that asked participants to reflect on difficult thoughts, feelings, and sensations. However, this is arguably an unavoidable consequence of efforts aimed at developing a better understanding the mental health and wellbeing of refugees, in turn informing efforts to support them.

Each of the four instruments demonstrated at least adequate levels of internal consistency in both sites, with both the PHQ-9 and MSPSS demonstrating very good levels. The current study indicates that the adapted and translated versions of the PHQ-9 in both sites demonstrates good construct validity (i.e. the PHQ-9 was measuring the appropriate theoretical construct). Owing to the fact that we did not have a ‘gold standard’ measure of depression administered by a trained mental health professional (e.g. a diagnostic interview), to analyse the PHQ-9’s construct validity we used a ‘known group’ approach based on that used by Bolton [47]. Specifically, we enlisted the support of community-based counsellors and support workers to identify people in the two refugee communities who were suspected of having high vs. low levels of depressive symptoms. In our analysis, we found that the individuals suspected of having high levels of depressive symptoms had significantly higher scores on the adapted version of the PHQ-9 in both sites, thereby indicating good construct validity of our adapted measure.

In our study, the concurrent validity of the adapted versions of the PHQ-9 was supported in both sites through the significant positive correlations that were noted with the CDES. The CDES has previously been used amongst Rohingya refugees in Bangladesh [37]. In that study, a larger number of reported daily environmental stressors was associated with higher levels of depressive symptomatology. These findings are consistent with the important role that displacement related and daily stress play in the emotional wellbeing of forcibly displaced people [17, 60]. The adaptation process for the CDES indicated that the 14 items of the original version were considered to be relevant for life in Kyangwali and Gihembe—with two new items being added for Kyangwali and one item added for Gihembe. Building on the work of Bolton and Tang [61], the addition of these items to the CDES was informed by rapid qualitative research that we undertook with the communities in Kyangwali and Gihembe [10].

The PHQ-9 has been widely validated and used on the African continent [36, 62–67]. Mwangi and colleagues [66] report on the validation of an adapted version of the PHQ-9 for use with Swahili speaking people living with HIV in the Kilifi County area of coastal Kenya in a study conducted by Nyongesa and colleagues [68]. The authors noted that “*Where appropriate, additional locally relatable examples were added such as ‘reading the Bible or Quran’ to item 7 of the PHQ-9 ‘trouble concentrating on things, such as reading the newspaper or watching television’*” [66]. This is similar to the changes that were made to the item 7 in Gihembe where the examples were changed to ‘*listening to the radio or caring for family*’.

The current study also provides evidence to support the convergent validity of the adapted versions of the SASCAT and the MSPSS. The levels of social capital (as assessed by the SASCAT) had significant positive correlations with each of the indices of perceived social support assessed by the MSPSS (including overall perceived social support as well as social support from significant others, family members and friends). To date, we are not aware of research that has used the MSPSS to assess perceived social support in adult refugees living in Rwanda or Uganda. Results from our recent rapid qualitative study suggest that the diverse make-up of Congolese refugees (in terms of ethnicity and language) in Kyangwali may be contributing to lower levels of perceived social cohesion, whereas in Gihembe the more homogeneous and close-knit nature of the population may be associated with less conflict between groups and higher levels of social support [10]. The larger geographic size of the Kyangwali settlement and wider dispersal of Congolese refugees may have implications for perceptions of social support when compared to the more densely inhabited Gihembe camp where people who can provide social support may be more readily available.

The study had some important potential limitations. Although there was scope to consider local idioms of distress [69, 70] during *Step B* of the three-stage procedure used to linguistically translate and culturally adapt the assessment instruments (see Table 1), had there been more time and resource, we could have focused more on identifying and assessing the idioms of distress or local syndromes experienced by the Congolese refugees living in Gihembe and Kyangwali. This has been highlighted by previous research as an important consideration in assessing mental health and wellbeing [71]. Qualitative research conducted with Kinande speaking people living in the Butembo in the North Kivu province of DRC identified local syndromes including *erisire* or *amutwe alluhire* (‘tired head’) [72]. Future research may wish to develop novel assessment instruments for measuring idioms of distress and local syndromes pertinent to the Congolese refugee communities in Kyangwali and Gihembe.

The participants recruited in the rapid validation phase in both sites led to an imbalance between genders, with more females participating in the study in both sites (Gihembe: 72 vs. 28; Kyangwali: 65 vs. 35). The purposive approach to the sampling of participants, which sought to recruit people on the basis that they identified as having either high or low levels of depressive symptomatology, may have inadvertently skewed the gender distribution. In Gihembe, in particular, females were markedly over-represented in the group adjudged to have high levels of depressive symptoms by the local informants (44 females vs. 6 males). The samples are not representative of the population as a whole, and as such the extent to which the findings from the current study generalize to the refugee population more widely in Gihembe and Kyangwali is unclear.

## Conclusion

The two studies reported in the current paper describe a pragmatic empirical approach to contextually adapting and validating mental health and wellbeing assessment instruments for use in complex humanitarian settings. Members of the local Congolese refugee communities were actively involved in our approach to maximise the relevance, understandability and acceptability of the translated and adapted items of four assessment instruments measuring dimensions relevant to mental health and wellbeing. The processes used in the current studies highlight the valuable opportunities that the adaptation of assessment instruments provide for proactively and sensitively engaging with communities to co-develop an approach to assessing mental health and wellbeing in meaningful and locally contextualised ways. The adapted versions of the PHQ-9, CDES, SASCAT and MSPSS will be used in the CoSTAR randomized controlled trial of a psychosocial intervention to address mental and social distress experienced by refugees in Rwanda and Uganda.

## Data Availability

The datasets used and/or analysed during the current study are available from the corresponding author on reasonable request.

## Abbreviations

CDES: Checklist for Daily & Environmental Stressors
CoSTAR: Community-based Sociotherapy Adapted for Refugees
DRC: Democratic Republic of Congo
MHPSS: Mental Health and Psychosocial Support
MSPSS: Multidimensional Scale of Perceived Social Support
PHQ-9: Patient Health Questionnaire-9
RCAG: Refugee Community Advisory Groups
SASCAT: The Shortened and Adapted Social Capital Assessment Tool
